# Ultrasonic modification of carbon materials for electrochemical capacitors

**DOI:** 10.1186/s11671-017-1842-1

**Published:** 2017-01-28

**Authors:** Bogdan I. Rachiy, Marian O. Nykoliuk, Ivan M. Budzulyak, Andrii I. Kachmar

**Affiliations:** Vasyl Stefanyk PreCarpathian National University, 57 Shevchenko Str., Ivano-Frankivsk, 76018 Ukraine

**Keywords:** Ultrasonic modification, Nanoporous carbon material, Electrochemical capacitor, Chemical treatment, Capacity, 81.05.Uw, 82.45.Gj, 82.47.Uv, 88.80.Fh

## Abstract

The paper is devoted to study the ultrasonic impact on the biomass of natural raw materials, which were used for the creation a nanoporous carbon material (NCM), which was used as electrode material for electrochemical capacitors (EC). The dry shells of apricot seeds were a feedstock, which were modified by the chemical treatment in the phosphoric acid and part of them were impacted by ultrasonic waves for 25 minutes. The NCM, which were obtained by carbonization at 550 °C, were modified by chemical treatment in the nitric acid. Thus, the different of modification NCM was obtained to compare their capacitance characteristics for EC. From experimental data we can do a conclusion, that ultrasonic modification and chemical treatment in nitric acidare improvecapacitance characteristics of NCM for EC.

## Background

The capacity of electrochemical capacitors are mainly determined by high surface are of electrode material and by appropriate pore size distribution [1, 2]. But even for optimal pore size it is not enough to create electrical double-layer (EDL), because notmore than 50% of surface area are taken part in charge/discharge processes [3].

The reasons, which prevent to create EDL are theavailability of graphite inclusions on the pore surface, which is not wetted with electrolyte, functional groups which are responsible for chemical interaction with the electrolyte, the viscosityof the electrolyte and etc. it means that in general energy barriers appears, which were overcome by ions for creation of EDL on the pore surface.

Obviously, there are another external factors, which have influence on the surface area, electrical and energetic states, and can stimulate the creation of additional EDL surface. Ultrasonic, laser radiation, electromagnetic field can belike external factors, which can be used on the first stage of carbon obtaining and modification or when it is used in EC [4, 5].

There are some papers, where only some aspects are mentioned in this problem, they bring only a fragmented information and do not show us real image of how this factors influence on charge/discharge processes in EC [6, 7]. This paper is devoted to study the mechanisms of EDL creation by ultrasonic modification.

## Methods

The methods from paper [8] were used to get NCM, where the main precursor was phosphoric acid. We receive series of samples which half were modified by ultrasonic waves for 25 minutes using a dispersant UZDN-A (operating frequency 22 ± 1,68 kHz) at maximum capacity. Then, all samples were dried at 90 °C to constant weight and the carbonization of feedstock was carried at 550 °C. The part of them were modified by chemical treatment in nitric acid by stirring in a magnetic mixer for 3 hours. All samples were washed to a neutral pH and were dried to constant weight.

As a result, four series of electrode material were gotten:

1. Series 1 – processing in the phosphoric acid;
2. Series 2 – processing in the phosphoric acid with ultrasonic modification for 25 minutes;
3. Series 3 – processing in the phosphoric and nitric acid;
4. Series 4 - processing in the phosphoric acid with ultrasonic modification for 25 minutesfollowed by processing in nitric acid;

The electrodes of the investigated EC were formed in the form of lamel from the mixture of:<NCM>:<CA > = < 3>:<1>, where CA is conductive additive. The resulting symmetrical electrodes were seeped by electrolyte, were separated by sealant and placed into the 2-electrodes cell with typical size “2525”, where after it was sealed. The 33% KOH was used as an electrolyte.

The research of electrochemical properties of EC were done by galvanostatic and potentiodynamic cycling. The measurement was made on the complex AUTOLAB PGSTAT12 of the company “ECO CHEMIE” (Netherlands) and stocked with firmware GPES and FRA-2. The galvanostatic measurement was made in the voltage range 0 – 1 V and current of charge/discharge was changed from 10 to 200 mA in increments of 10 mA.

The capacitance was calculated by the equation: *C* = *2I* · *t*_*d*_/[(*U*_*m*_-Δ*U*) · *m*], where *I* is current of charge/discharge, *t*_*d*_ is a time of discharge, *U*_*m*_ is a maximum voltage, Δ*U* is a voltages drop of short circuit of discharge circuit, m is a weigh.

Specifications of NCM porous structure (it means surface area and pores total volume) was determined on the basis of analysis of adsorption/desorption isotherms of nitrogen at temperature of its ebullition (-196 °C), received from QuantachromeAutosorbNova2200e.

## Results and discussion

**Fig. 1:**
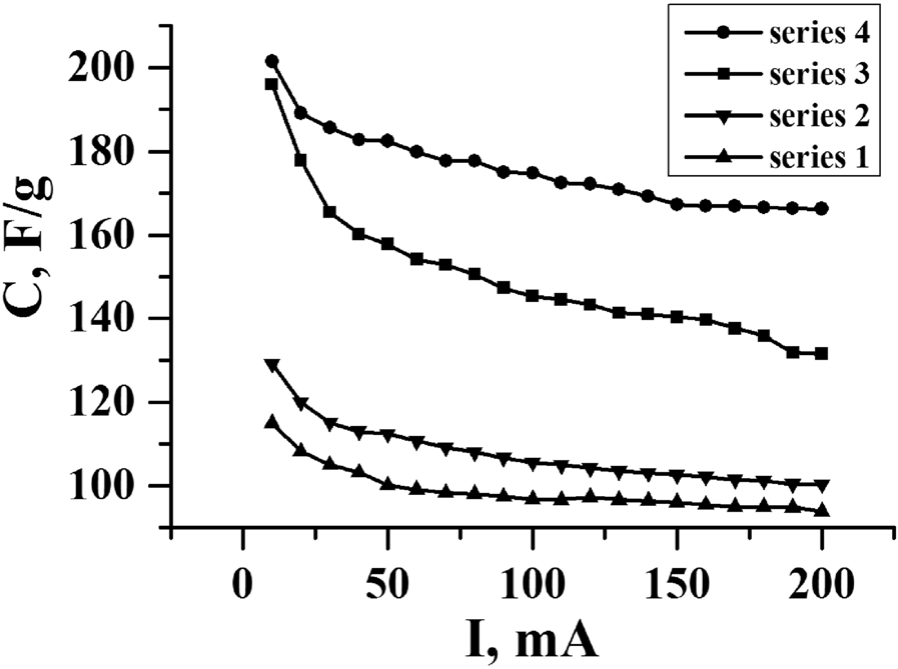
The dependence of NCMcapacity C (F/g) from charge/discharge current I (mA) for different series

From the analysis of the dependence of C from I (Fig. 1) by using different methods of modification of carbon material, we can make a conclusion that chemical treatment in nitric acid makes it possible to enhance the cell capacity of 1,5-2 times (Fig. 1, series 1). In each case, with and without chemical treatment in nitric acid, ultrasonic modification as a result also shows significant improvement of capacitance characteristics by 10-25%.

In particular, as shown in Fig. 1, even at high currents charge/discharge it shows that samples with ultrasonic modification (series 2 and series 4) have more stable and straight curves, and the smallest decline is observed with increasing current capacity from 10 to 200 mA. This may be due to the fact that under the ultrasonic modification the phosphoric acid filled structural pore in the natural material and the carbonization formed carbon material with appropriate porous microstructure, which provides these properties.

The capacity is greatest for series 4. The decrease of capacity value is with each increase of current charge/discharge, and its average value for series 4 is C = 175 F/g throughout the period of the period of the current charge/discharge 10-200 mA (the maximum value is C = 202 F/g at I = 10 mA).

**Fig. 2:**
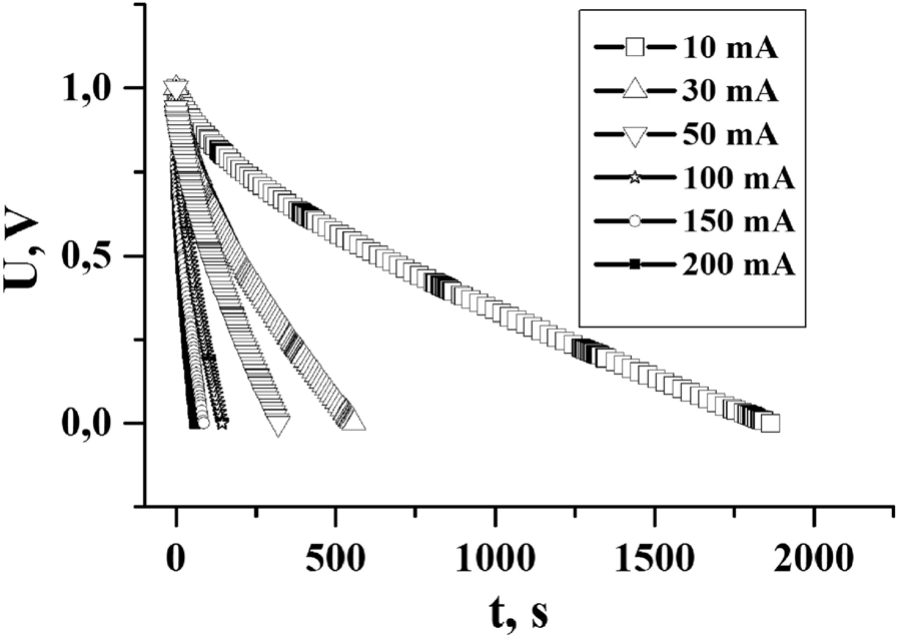
Some discharge curves of EC, which are based on electrode material from series 4 at constant discharge currents (10,30,50,100,150,200 mA)

It is also worth noting that the EC based on NCM, which was obtained by chemical treatment in phosphoric acid, can be maintained at sufficiently high values of current charge/discharge (>150 mA). The charge/discharge curves of EC based on NCM series 4 are shown in Fig. 2.

As shown in Fig. 2, the discharge time was 1 minute at a constant discharge current at 200 mA, which is quite high for this type of electrochemical capacitor [9] and provides an opportunity to conduct research at a sufficiently high current values.

The results indicate that at the ultrasonic modification with chemical treatment in phosphoric acid of natural material (shells of apricot seeds) and processes in nitric acid of NCM we have an effective electrode material for EC. This allows you to enhance energetic properties of NCM and to increase EC capacity.

The chemical treatment in nitric acid cleans the porous structure of NCM from natural resins and that how it increases the number of effective pore, which are involved in the creation of electrical double-layer (EDL).

**Fig. 3:**
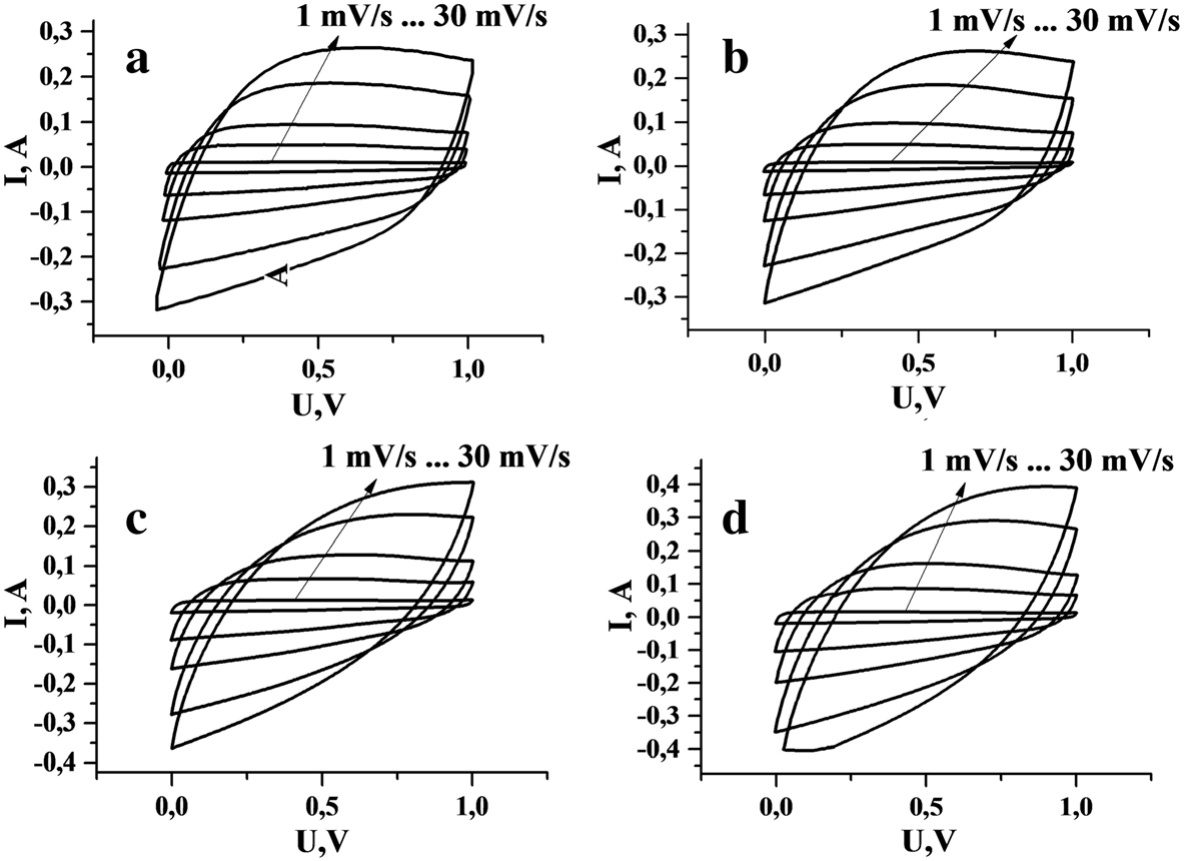
Potentiodynamic curves of EC, which are taken at different speed scan (s = 1; 5; 10; 20; 30 mV/s) for the corresponding NCM as an electrode material: a) series 1, b) series 2, c) series 3, d) series 4

According to the potentiodynamic research in field of potential at 0-1 V, the cyclical potentiodynamic curves are shown in Fig. 3. At low speeds (1 mV/s and 5 mV/s) the differences between EC cells with different NCM are observed in the form of curves. This approximate rectangular shape is typical for EC using an aqueous electrolyte [9]. The characteristics of capacitance are close to the values which were obtained from galvanostatic research under the same current. According to the comparing voltammetrycurves (scanning at speed 1 mV/s, 5 mV/s and 10 mV/s), there is a little peak for all samples at potential 0,8… 1 V, it means, that the material is provided mainly negative ions of the electrolyte (- OH groups) [3]. Even for speeds at 20 mV/s and 30 mV/s we see the differences in the shape of curves between samples. The series 3 and 4, which were modified by chemical treatment in nitric acid (Fig. 3 c, d), their curves are “leaf” shape look, with pronounced curves without peaks. This form, but slightly, have seen for series 4 (Fig. 3 b), which were subjected by ultrasonic modification in phosphoric acid. This indicates that such processing of materials gives a result, where is more porous structure, which also participate in the formation of EDL and according to this, we can see such a modification in the potentiodynamic curves form at high speed scanning.

**Fig. 4:**
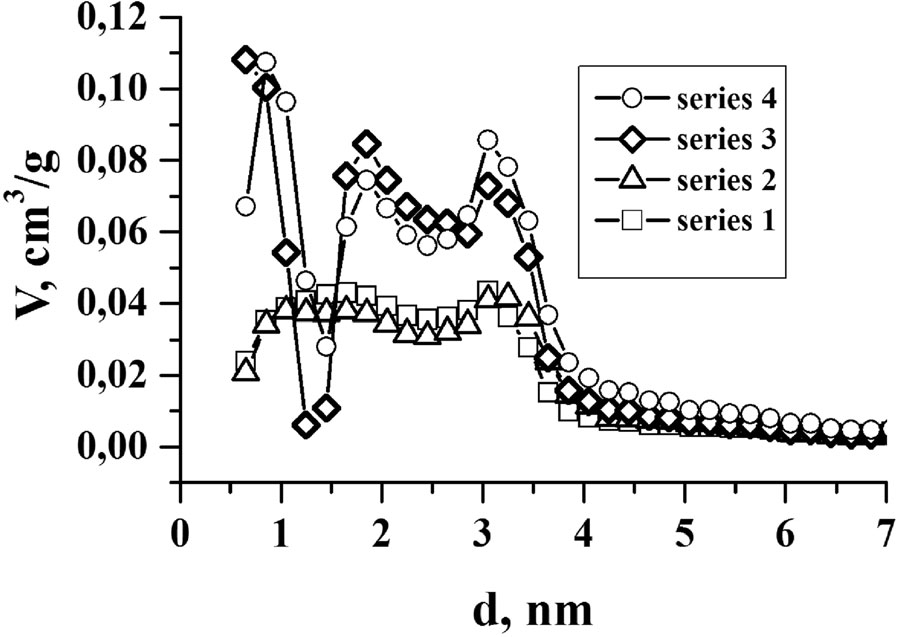
The dependence of pore volume *V* (cm^3^/g) from their diameter *d* (nm) for series 1,2,3,4

As evidence, the ultrasonic and chemical modification affect the formation of NCM and porosity measurement were done. The dependence of pore volume *V* (cm^3^/g) from their diameter *d* (nm) is shown in Fig. 4, where we can do the conclusion, that inthe charge/discharge processes of EC, mainly, mesopores take part in EDL formation.

As shown in Fig. 4, the NCM, which was modified by chemical treatment in nitric acid (series 3 and series 4), have a greater specific volume of micro and mesopores in the range of diameters from 1 … 4 nm. This suggests that the chemical processing allows us to obtain highly porous material and taking into account the results of galvanostatic research, it improves their capacitance characteristics for use in the EC. We can also note the presence of micropores with diameter of 0.6 … 2 nm in these samples (series 3 and series 4), which probably also plays a role in improving EDL properties for EC.

The ultrasonic modification makes minimal changes in formation of NCM that has an effect in enhancing the capacitance of EC.

**Fig. 5:**
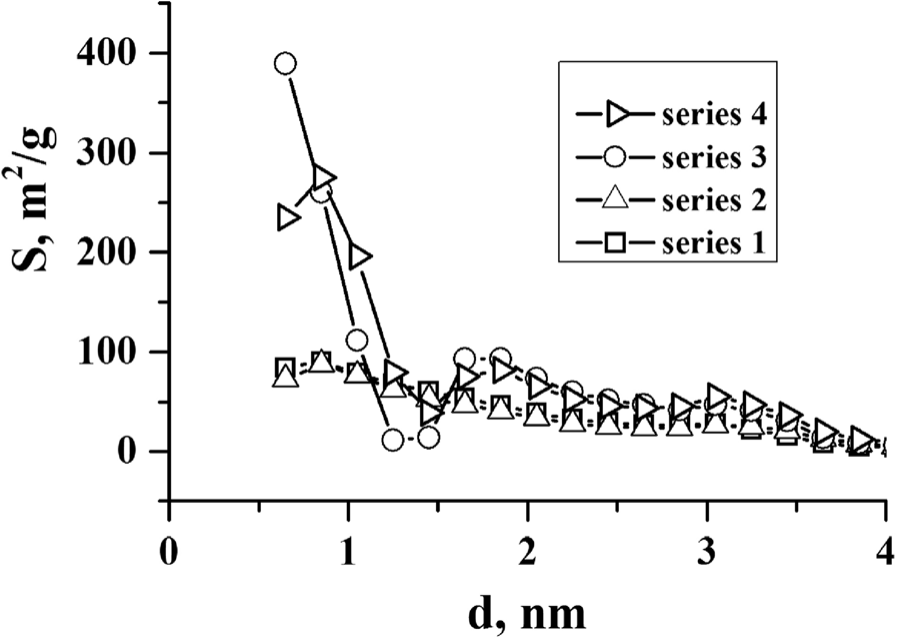
The dependence of surface area S (m^2^/g) from their diameter *d* (nm) for series 1,2,3,4

The typical pattern is observed in the dependence curves of surface area *S* (m^2^/g) from diameter *d* (nm) in Fig. 5.

The increasing of surface area is observed for samples that were chemically modified in nitric acid, and vast area are occupied by micropores. The ultrasonic modification provides only to expand the range of average diameters of micro- and mesopores.

In the Table 1, is presented calculated surface area *S*_*BET*_(m^2^/g) by BET theory, the total pore volume *V*_*total*_ (cm^3^/g) and average pore diameter *d*(nm) for 1,2,3,4 series.

**Table 1:** Calculated surface area *S*_*BET*_ by BET theory, the total pore volume *V*_*total*_ and average pore diameter *d* (nm) for different series

Series	1	2	3	4
*S*_*BET*_, m^2^/g	950	875	1660	1880
*V*_*total*_, cm^3^/g	1	0,8	1,3	1,4
*d*, nm	4	3,5	3	3,5

According to the analysis of the data in the Table 1, we can conclude that ultrasonic and chemical modification in nitric acid gives an opportunity to get the average values pore diameter values of the smaller values and thus to enhance capacitance characteristics of EC. Also it shows that the chemical treatment in nitric acid makes it possible to increase surface area of 2 times (series 3 and series 4). The ultrasonic modification gives us the same result for samples, which were processed in nitric acid, where the surface surface area of the series 4 is greaterthan series 3up to 200 m^2^/g, and the average pore diameter is about the same. The highest energy performance was showed by EC, which based on the series 4, where the combination of ultrasonicand chemical modification in nitric acid, we see that the surface area has 2 times greater value from the original series 1, the specific pore volume is greater and the presence of average pore diameter is ≈ 3 nm. It can be concluded that the optimal energy properties forNCM are with a high specific pores volume (*V*_*total*_, cm^3^/g), surface area (S_BET_, m^2^/g) and the small value of the average pore diameter*d* (nm).

As a result, we can say that ultrasonic modification in phosphoric acid of natural raw materials and chemical treatment in nitric acid enhance energy properties of NCM for EC. Such an approach in the creation of new methods of modification is important for research and allows to get better capacitance characteristics of EC.

## Conclusions

The chemical treatment of natural materials (shells of apricot seeds) in phosphoric acid allows us to get the optimal NCM electrode material for EC, which can be operated at high speeds of charge/discharge current at 150-200 mA.

The ultrasonic modification in phosphoric acid at frequency of 22 kHz for 25 minutes provides significant performance improvements of EC about 25%. Also worth noting that chemical treatment of stirring in nitric acid for 3 minutes gave us a significant improvement of NCM capacitance. It gives us to upgrade the EC in 1,5-2 times. Such methods of processing materials for EC electrodes are environmentally safe and easy to use, which is especially cost-effective.

## Abbreviations

BET: Brunauer-Emmett-Teller theory
EC: Electrochemical capacitors
EDL: Electrical double-layer
NCM: Nanoporous carbon material
